# The Pet-Specific Reasons for Living Scale: development, validation, and associations with suicide-related outcomes

**DOI:** 10.3389/fvets.2026.1889983

**Published:** 2026-08-06

**Authors:** Shelby E. McDonald, Camie A. Tomlinson, Stacey L. Freedenthal, Jada Ford, Hannah Van Buiten, Lori R. Kogan, Nicole Nicotera, Jennifer Currin-McCulloch, Amelia Malone

**Affiliations:** 1 Colorado State University School of Social Work, Fort Collins, CO, United States; 2 Kent School of Social Work and Family Science, University of Louisville, Louisville, KY, United States; 3 Graduate School of Social Work, University of Denver, Denver, CO, United States; 4 Independent Researcher, Kailua, HI, United States; 5 Department of Clinical Sciences, College of Veterinary Medicine and Biomedical Sciences, Colorado State University, Fort Collins, CO, United States

**Keywords:** companion animals, human–animal bond, measurement invariance, psychometrics, reasons for living, suicidal ideation, suicide prevention

## Abstract

**Background:**

Companion animals may represent salient reasons for living, yet existing measures do not assess pet-related commitments. This study developed and validated the Pet-Specific Reasons for Living Scale (Pet-RFL) and examined its associations with mental health and suicidality.

**Method:**

Data were drawn from 962 U.S. adults completing an online survey. Sixteen Pet-RFL items, modeled on the Reasons for Living Inventory, were evaluated using exploratory factor analysis in a random subsample and confirmatory factor analysis in the full sample. Measurement invariance was tested across sociodemographic groups. Construct validity was examined via associations between each Pet-RFL factor score and depression, anxiety, stress, suicidal ideation, lifetime suicide attempt history, social support, and RFL domains.

**Results:**

A two-factor structure was supported, reflecting pet-related belongingness/responsibility and harm-avoidant obligation/concern. Both factors demonstrated excellent internal consistency and strong measurement invariance. Adjusting for covariates, higher belongingness/responsibility scores were associated with lower suicidal ideation, fewer depressive and stress symptoms, reduced odds of lifetime suicide attempt history, and greater survival/coping beliefs and moral objections as measured by the RFL. In contrast, when adjusting for covariates, higher harm-avoidant obligation/concern scores were associated with greater suicidal ideation, more depressive and stress symptoms, higher likelihood of lifetime suicide attempt history, and greater responsibility to family and fear of suicide and lower moral objections as measured by the RFL.

**Conclusion:**

The Pet-RFL is a psychometrically supported measure capturing distinct pet-related reasons for living with differing associations to suicidality. Further, this study preliminarily distinguishes between belonging-based and harm-avoidant reasons for living related to companion animal relationships, highlighting the complex roles companion animals may play in human wellbeing, multispecies caregiving, suicidality, and veterinary social work practice.

## 1 Introduction

Suicide is a global public health crisis. Each year, more than 700,000 people die by suicide worldwide, making it one of the leading causes of preventable death (1, 2). Globally, suicide is the third leading cause of death among people aged 15–29 years, and in the United States it ranks 11th overall (1–3). Although suicide occurs across populations, disparities in risk are substantial. In the United States, non-Hispanic American Indian and Alaska Native (AI/AN) populations have the highest age-adjusted suicide rates of any racial/ethnic group, with especially high rates among non-Hispanic AI/AN males ages 15–34; elevated risk is also observed among military veterans, people who are incarcerated, and sexual and gender minority youth (1–4). Older adults, particularly those experiencing social isolation or chronic illness, also face increased vulnerability (5, 6). Recent surveillance data further indicate that suicide attempts have risen most sharply among Black youth, even as corresponding rates have declined among their White peers (7).

Research has identified several protective factors that reduce suicide risk, including reasons for living (i.e., life-preserving beliefs and responsibilities) (8, 9); social support, connection, and belongingness (10); problem-solving and adaptive coping skills (11); hope, purpose, and resilience (12); religious and spiritual beliefs (13); reduced access to lethal means (14); and access to mental health services (15). Additionally, research has identified the potential role of companion animals as salient sources of meaning, responsibility, and suicide-related protection (16–20). Building on this work, the current study advances understanding of the human–animal bond in reasons for living through the development and psychometric evaluation of the Pet-Specific Reasons for Living Scale (Pet-RFL). Specifically, we assessed the factor structure, internal consistency, construct validity, and measurement invariance of the newly developed Pet-RFL, and examined its associations with suicidal ideation, depression, stress, and anxiety.

### 1.1 Protective beliefs, commitments, and the Reasons for Living Inventory

Protective factors that reduce suicide risk span demographic, behavioral, psychological, and social domains (12, 21, 22). Attitudes and beliefs about suicide, as well as perceived responsibilities that deter a person from acting on suicidal thoughts, are also considered important protective processes. A leading measurement framework, the Reasons for Living Inventory [RFL; (8)], was developed to assess beliefs, commitments, and fears that counterbalance suicidal urges, including survival and coping beliefs, responsibility to family, child-related concerns, moral objections, fear of suicide, and fear of social disapproval (8). The RFL and short-form variants of the scale have been used with adolescents and adults across the lifespan, with higher scores consistently associated with lower suicidal ideation and reduced attempts (23, 24).

Importantly, reasons for living are shaped by culture, developmental stage, and lived experience. For example, cultural consensus modeling with Black adolescents identified racism, poverty, and bullying as salient reasons for suicide, whereas family, community belonging, and hope for the future were emphasized as reasons for living (7). In adolescents more broadly, future-oriented goals and peer relationships commonly surface as reasons for living (25, 26). Among older adults, studies suggest that spiritual beliefs, social support, responsibilities toward family, and contact with pets help mitigate suicidal thoughts and behavior (27, 28). For people experiencing structural marginalization or serious mental illness, human and companion-animal relationships can provide stability and emotional grounding that strengthen coping and reduce loneliness (29, 30). Collectively, these findings underscore that reasons for living are not uniform, highlighting the need for measurement approaches that capture diverse relational and contextual commitments across populations, including commitments embedded within multispecies caregiving systems.

### 1.2 Companion animals as potential protective factors

Qualitative and mixed-methods studies consistently describe pets as attachment figures that provide acceptance, belonging, and emotional comfort (31, 32). Research also indicates that pets may reduce feelings of thwarted belongingness and perceived burdensomeness (33, 34), both of which are associated with suicidal behavior, per the Interpersonal Theory of Suicide (35). In numerous studies across diverse populations, individuals have reported choosing to stay alive because of their animals. Survivors of intimate partner violence have recounted foregoing suicide attempts because they could not abandon their pets (36). Older adults credit pets with mitigating loneliness, providing purpose, and serving as a source of companionship and comfort (6). LGBTQ+ youth experiencing homelessness identify pets as a primary source of consistency, companionship, and responsibility (37, 38). Adults with autism report that interactions with a dog (e.g., tactile interactions, greetings, walking) improve mood and contribute to lessened suicidality (16). Veterans with posttraumatic stress disorder frequently credit service dogs with increased daily structure and meaning; case and cohort reports even describe dogs interrupting suicidal behavior (20, 39). Lastly, individuals living with borderline personality disorder also describe pets as providing meaning and structure that can mitigate suicidal impulses (40).

Multiple mechanisms have been proposed for these protective effects. Attachment and companionship cultivate felt connection; caregiving responsibilities foster obligation and purpose; and behavioral activation (e.g., walking, feeding, routines) can interrupt rumination, sustain engagement in life (6, 31, 32), and distract from suicidal thoughts (17, 41). In some cases, pets have provided comfort that shifted the trajectory of a suicidal episode (17). These findings suggest that pet-related reasons for living may emerge through both connection- and meaning-based processes as well as concern-centered beliefs related to caregiving, obligation, and companion animal welfare. A recent scoping review similarly concluded that companion animals may influence suicidality through multiple pathways, including attachment, caregiving responsibility, meaning, grief, and veterinary access-related stressors, underscoring the need for measures capable of distinguishing adaptive pet-related reasons for living from distress-linked obligation or welfare concerns (19).

### 1.3 Risks and complications of the human–animal bond

The human–animal bond is not uniformly protective and may influence suicidality through both protective and risk-complicating pathways. Pet loss can precipitate intense grief and has been linked to suicidal crises, especially among people with limited social support (5, 42). Caregiving burden, financial strain, housing restrictions, and occupational exposures (e.g., euthanasia-related stress in veterinary and shelter settings) can generate or exacerbate distress (43, 44). Rarely, stress due to pets' health or behavioral problems can exacerbate or even activate suicidal ideation (17). Additionally, having pets in the household may increase access to lethal means (e.g., veterinary medications) or be involved in “extended suicide” scenarios, in which a person kills an animal (or another dependent) while also taking their own life (45–47). These findings underscore that suicidality may also have direct implications for companion animal welfare and safety. A 2025 scoping review of 25 studies concluded that while many qualitative reports describe owner-perceived protection (e.g., connection, purpose, caregiving), observational studies show inconsistent associations between pet ownership and suicidal ideation or death (20); notably, one prospective cohort study found a positive correlation (*r* = 0.17) between time spent with one's dog and suicidal ideation, suggesting that the relationship may be bidirectional, context-dependent, or influenced by third variables such as social isolation or depression severity (16, 48).

### 1.4 The current study

National data suggest that companion animal ownership may now be more prevalent than parenthood and may even rival rates of partnership or marriage in some age groups (49–55), indicating that pets represent an increasingly common source of meaning and responsibility. While Linehan and colleagues' original *RFL* (1983) was developed to assess beliefs, fears, and responsibilities that protect against suicidal behavior, it does not assess the role that animals may play in supporting wellbeing and purpose for many individuals. This omission may represent a significant gap in both research and practice. Clinicians may overlook a client's most salient sources of meaning and responsibility, while researchers are unable to evaluate the unique or incremental protective value of pet-related commitments relative to established RFL domains, including their relevance within multispecies caregiving and behavioral health systems.

Informed by the Reasons for Living Inventory, the present study developed and validated the Pet-Specific Reasons for Living Scale (Pet-RFL) to examine whether commitments related to companion animals function as reasons for living. In doing so, it advances theory by broadening the conceptualization of reasons for living to include multispecies sources of purpose and responsibility, and it advances practice by offering clinicians a tool to assess dimensions of meaning and obligation that are often overlooked yet deeply salient for many clients.

## 2 Methods

### 2.1 Participants

Data were collected from 1,147 U.S. adults (aged 18 years and older) who participated in an online survey administered between June 2–3, 2025. Participants were recruited via Prolific, a licensed online research platform that has been shown to yield high-quality behavioral health data and more diverse samples than many convenience online sources (56). Respondents received $7.50 for survey completion, with an additional incentive of up to $3.00 for completing pet-related items. Informed consent was obtained on the first survey page; individuals who declined consent were exited from the study. All surveys were administered in English. Study procedures were reviewed and approved by the Colorado State University Institutional Review Board. The final analytic sample used in psychometric analyses (*n* = 962) is described below, following the exclusion of missing data and subsample selection procedures. The sample included individuals with diverse relationship and family structures, including participants who were single and partnered and those with and without children.

### 2.2 Measures

#### 2.2.1 Reasons for Living Inventory

The Reasons for Living Inventory [RFL; (8)] is a 48-item self-report measure that assesses beliefs and values considered to protect against suicide. Items are rated on a 6-point Likert scale ranging from 1 (“not at all important”) to 6 (“extremely important”). The RFL yields six subscales—Survival and Coping Beliefs, Responsibility to Family, Child-Related Concerns, Fear of Suicide, Fear of Social Disapproval, and Moral Objections, as well as a total score. Higher scores indicate greater endorsement of reasons for living. The RFL has demonstrated strong reliability and validity across clinical and non-clinical samples [e.g., (8, 85, 86)]. In the current study, internal consistency of the RFL total score and all six subscale scores were good to excellent (ω = 0.83–0.97).

#### 2.2.2 Pet-specific RFL items

To capture the role of companion animals as reasons for living, we modeled our pet-specific scale on the RFL. Item development followed established best practices for measure adaptation and extension. Specifically, a team of faculty with expertise in human–animal interaction (HAI), social work, grief, and suicide prevention generated an initial pool of items informed by prior qualitative findings [e.g., (34)] and the broader HAI literature. Draft items were modeled directly on the structure and phrasing of the original RFL (e.g., retaining the implied stem “I would not kill myself because…”) to ensure conceptual continuity. Pet-specific content emphasized themes such as responsibility for a pet's care and concern for the pet's wellbeing if the individual were no longer present. Items were intentionally designed to capture attachment-, responsibility-, and obligation-based pathways through which pets may function as reasons for living. Through iterative review and consensus among the faculty team, items were refined for clarity, contextual relevance, and theoretical fit with the RFL framework. This process resulted in a final set of 16 items (see Table 1), which was administered with the original RFL Inventory in the current study.

**Table 1:** Frequencies of endorsement of each original and collapsed response scale option for the pet-RFL items (*N* = 962).

Pet-RFL item	Original response scale					Collapsed response scale			
	1	2	3	4	5	6	1–3	4–5	6
1. I have a responsibility to my pet(s).	16	15	30	151	286	464	61	437	464
2. I have a commitment to my pet(s).	17	16	29	158	305	437	62	463	437
3. My pet(s) might believe I do not love them.	195	103	106	183	188	187	404	371	187
4. My pet(s) depend upon me.	19	18	30	122	279	494	67	401	494
5. My pet(s) need me.	19	14	29	125	280	495	62	405	495
6.I love and enjoy my pet(s) too much and could not leave them.	27	15	44	156	276	444	86	432	444
7. It would hurt my pet(s) too much and I would not want them to suffer.	58	41	60	163	262	378	159	425	378
8. I want to continue making memories with my pet(s).	35	24	56	163	277	407	115	440	407
9. It would not be fair to leave my pet(s) for others to take care of.	45	45	76	184	290	322	166	474	322
10. The effect on my pet(s) could be harmful.	44	57	78	189	258	336	179	447	336
11. I worry about what would happen to my pet(s) if I were gone.	39	35	47	126	279	436	121	405	436
12. The companionship of my pet(s) makes life more meaningful.	17	17	21	138	290	479	55	428	479
13. My pet's(s') quality of life would be negatively impacted if I was gone.	32	36	60	186	296	352	128	482	352
14. I would let my pet(s) down if I chose to not be here anymore.	47	41	57	172	281	364	145	453	364
15. I don't want my pet(s) to end up homeless or in a shelter.	41	23	35	108	212	543	99	320	543
16. My pet(s) might feel abandoned.	48	43	50	148	246	427	141	394	427

Items were developed for the Pets, Attachment, and Mental Health Study to assess reasons for living that specifically reference responsibilities and emotional bonds with companion animals. Participants were instructed: “*Many people have thought of suicide at least once. Others have never considered it. Whether you have considered it or not, we are interested in the reasons you would have for not acting on suicidal thoughts if they did arise or if someone were to suggest it to you. On this page are reasons people sometimes give for not acting on suicidal thoughts. We would like to know how important each of these possible reasons would be to you at this time in your life as a reason to not kill yourself.”* Items were rated on a 6-point scale originally: 1 = not at all important, 2 = quite unimportant, 3 = somewhat unimportant, 4 = somewhat important, 5 = quite important, 6 = extremely important.

#### 2.2.3 Depression, anxiety and stress

We used the Depression, Anxiety and Stress Scale (DASS-21) to assess symptoms of depression, anxiety and stress among participants (57, 58). The DASS- 21 consists of three self-report subscales, each containing seven items assessing the frequency of depression, anxiety, and stress symptoms. These items are scored on a four-point Likert scale from “never” to “almost always.” For this study, we calculated the subscale scores for depression, anxiety and stress by summing the item scores and then multiplying those scores by two for each subscale. Because the original cut-off scores for severity (normal, mild, moderate, etc.) were created using the DASS-42 item scale (57), multiplying the DASS-21 scores by two allows interpretation using the established DASS-42 severity ranges. DASS-21 scoring indicates that depression, anxiety, and stress scores are considered to be within a normal severity range if they fall between 0–9, 0–7, and 0–14, respectively. Mild scores range from 10–13, 8–9, and 15–18. Moderate scores range from 14–20, 10–14, and 19–25. Severe scores range from 21–27, 15–19, and 26–33; and extremely severe scores are greater than or equal to 28, 20, and 34, respectively (58). For this study, internal consistency was excellent for depression (ω = 0.93), anxiety (ω = 0.89), and stress (ω = 0.91).

#### 2.2.4 Social support

Perceived social support was assessed using the Multidimensional Scale of Perceived Social Support (59). The MSPSS is a short scale of 12 items designed to measure an individual's perception of support from friends, family and significant other(s). Items are ranked on a seven-point Likert scale ranging from “very strongly disagree” to “very strongly agree.” For this study, a total perceived support score was calculated as the mean of all 12 items. Internal consistency of the MSPSS total score was excellent (ω = 0.95).

#### 2.2.5 Suicidality

##### 2.2.5.1 Beck scale for suicide ideation (BSS)

Suicidal thinking was evaluated using the Beck Scale for Suicide Ideation [BSS; (60)] to assess the severity of suicidal ideation over the past week, including suicidal desire, intent, and planning. Items are rated on a 0 to 2 scale. The first 5 items function as screener items, and individuals who answer “0” to items 4 and 5 are routed to items 20 and 21, indicating the absence of current suicidal desire. For this study, suicidal ideation was calculated by summing the 19 scored items reflecting past-week suicidal ideation severity. Items 20 and 21 are generally used for clinical information regarding an individual's past suicide attempts and therefore were not included in the total score. Scores range from 0 to 38, with higher scores indicating greater suicidal ideation severity (60). Internal consistency of the BSS total score was good (ω = 0.87).

##### 2.2.5.2 Suicidal ideation attributes scale (SIDAS)

We used the Suicidal Ideation Attributes Scale (SIDAS) to assess the intensity of suicidal ideation in the past month, including frequency, controllability, closeness to attempt, distress associated with suicidal thoughts, and impact on daily functioning (61). The SIDAS consists of 5 items, each measured on a 10-point Likert scale, with higher scores reflecting more intense suicidal thoughts. For this study, total scores were summed to calculate a score ranging from 0 to 50. To improve quantifiability, we modified the wording of Item 1 to say, “On a scale from 0 to 10, where 0 means “Never” and 10 means “Always,” how often in the past month have you had thoughts about suicide?” Internal consistency of the SIDAS total score was good (ω = 0.83).

##### 2.2.5.3 Attempts

Lifetime suicide attempt history was assessed using a single item from the Beck Scale for Suicide Ideation [BSS; (60)], which asks whether the participant has ever attempted suicide. Responses were recorded on a 3-point scale (0 = never attempted, 1 = one attempt, 2 = two or more attempts) and were recoded into a binary variable indicating no lifetime attempt (0) vs. any lifetime attempt (1).

#### 2.2.6 Covariates

We included sociodemographic covariates in conditional construct validity models to account for potential confounding by sociodemographic characteristics. We included gender modality (0 = cisgender man or cisgender woman, 1 = minoritized gender), relationship status (0 = single, 1 = in a relationship), child status (0 = no children, 1 = has child(ren)), age (continuous), race/ethnicity (0 = White/non-Hispanic, 1 = Black/non-Hispanic, Hispanic, or another minoritized racial/ethnic group), and pet type (dummy-coded: cat only, dog only, and cat and dog = reference group). For measurement invariance analyses, we examined gender modality/identity (cisgender woman, cisgender man, minoritized gender identity), race/ethnicity (White/non-Hispanic, Hispanic, Black/non-Hispanic, and other race/ethnicity), age (emerging adulthood: 18–25 years; early adulthood: 26–34 years; middle adulthood: 35–64 years; older adulthood: 65+ years), and pet type (cat only, dog only, cat and dog). Invariance based on relationship status and child status were assessed across only two groups (single/in a relationship and no children/has children).

### 2.3 Statistical analysis

Initial data cleaning was conducted in SPSS and all descriptive and bivariate statistics were conducted in Mplus. We used Mplus to conduct the EFA, CFA, invariance testing, and validity testing. All 16 Pet-RFL items were treated as ordered categorical variables using the weighted least squares mean and variance adjusted (WLSMV) estimator, which is comparable to a graded item-response theory model. The original items were rated on a 6-point scale; however, low frequencies in some response categories (e.g., low endorsement of importance) led to empty cells in the bivariate table during invariance testing causing estimation issues for the WLSMV estimator. Therefore, we recoded all items into 3 categories: not at all important/quite unimportant/somewhat unimportant, somewhat important/quite important, and extremely important.

We conducted EFA of the Pet-RFL items in Mplus in a small subsample to determine whether an alternative model could be identified (30%, *n* = 299). Based on the conservative recommendation of an item-to-respondent ratio of 1:20 (62), a minimum sample size of 320 participants was indicated. We therefore randomly selected approximately 30% of the sample (*n* = 299) for the EFA. We used geomin (oblique) rotation to include correlations between factors and an iterative, model-building strategy to increase the number of factors tested until additional factors no longer meaningfully improved the model fit. Model fit for each solution was assessed using multiple statistics, including the Kaiser-Guttman rule of retaining factors with eigenvalues > 1.0 (63), root mean square error of approximation (RMSEA) < 0.08, and comparative fit index (CFI) and Tucker-Lewis index (TLI) values > 0.90 (64). We also used the Mplus difference tests between models to determine if the added model complexity of an additional latent factor significantly improved model fit, retaining the additional factor if *p* < 0.05. We additionally considered the magnitude, significance, and cross-loading of item factor loadings and the overall interpretability of the factors in selecting the best-fitting EFA model.

Next, we tested whether the EFA-identified model would hold when applying the additional restrictions of the CFA (e.g., fixed cross-loadings) using the full sample. There are limitations to using the full-sample after the EFA (65); however, we chose to conduct the CFA with the full sample so that we could compare the EFA-informed model with theoretically informed CFA models and have sufficient group-sizes for invariance testing. These competing CFA models were selected to evaluate increasingly specific representations of the Pet-RFL, ranging from a single global pet-related reasons-for-living construct to empirically derived and theory-informed multidimensional structures. The four factors for the CFA were informed by the interpersonal psychological theory of suicide (35, 66) and research on reasons for living (67, 68). We compared the following in separate analyses: (a) a unidimensional model of all Pet-RFL items, (b) the two-factor EFA-informed model, and (c) a four-factor model that differentiated reasons for living based on responsibility/obligation to pets, pet dependence/welfare risk, emotional attachment and meaning, and anticipated emotional harm to pets. We also tested a higher-order four-factor model of a single overall pet-specific reason-for-living factor. We compared the fit of competing models using the difference test in Mplus and evaluated overall model fit using the same model fit indices as the EFA. Using the final 2-factor, EFA-informed model, we conducted measurement invariance tests across gender modality, race/ethnicity, relationship status, child status, age, and pet type.

Lastly, we conducted construct validity testing by examining associations between Pet-RFL factors and mental health (i.e., DASS-21), suicidal ideation (i.e., BSS, SIDAS), suicide attempt history, and the original RFL subscale scores.

## 3 Results

### 3.1 Descriptives

Our total analytic sample included 962 adults. Descriptives of the sample are included in Table 2. Inter-item correlations between all 16 Pet-RFL items are presented in Table 3. The Pet-RFL items and distribution of response options are shown in Table 1 for the original response scale and the collapsed response scale used in our analyses.

**Table 2:** Descriptive statistics for the full sample (*N* = 962).

Variable	*M/*#	*SD/*%
Age (*n* = 954)	45.64 years	16.11
18–25 years	130	13.60%
26–34 years	247	25.90%
35–64 years	445	46.60%
65+ years	132	13.80%
Gender Modality (*n* = 940)		
Cisgender woman	452	48.10%
Cisgender man	425	45.20%
Gender minority	63	6.70%
Sexual Orientation (*n* = 943)		
Straight	779	82.60%
Bisexual/Pansexual	97	10.30%
Lesbian/Gay	46	4.90%
Asexual/Demisexual	10	1.10%
Queer	10	1.10%
Two-Spirit	1	0.10%
Race/Ethnicity (*n* = 932)		
Hispanic	118	12.70%
Non-Hispanic/White	590	63.30%
Non-Hispanic/Black	108	11.60%
Other/Multi-racial	116	12.40%
Relationship Status (*n* = 947)		
Single	360	38.00%
In a relationship	587	62.00%
Pet Type (*n* = 945)		
Cat only	225	23.80%
Dog only	420	44.40%
Cat and Dog	300	31.70%
Has child(ren)? (*n* = 947)		
No	318	33.60%
Yes	629	66.40%
BSS total score	2.38	5.33
SIDAS total score	7.13	10.35
Suicide attempt ever (*n* = 958)		
No	759	79.20%
Yes	199	20.80%
RFL mean score	4.46	0.88

**Table 3:** Correlations between pet-RFL items.

Item	1	2	3	4	5	6	7	8	9	10	11	12	13	14	15	16
1	–															
2	0.87	–														
3	0.31	0.30	–													
4	0.75	0.75	0.34	–												
5	0.78	0.80	0.30	0.84	–											
6	0.67	0.68	0.36	0.65	0.69	–										
7	0.58	0.57	0.45	0.53	0.56	0.56	–									
8	0.57	0.60	0.28	0.55	0.61	0.66	0.53	–								
9	0.49	0.51	0.34	0.50	0.51	0.51	0.57	0.47	–							
10	0.50	0.50	0.47	0.51	0.52	0.52	0.62	0.45	0.54	–						
11	0.59	0.60	0.36	0.64	0.63	0.59	0.59	0.49	0.58	0.60	–					
12	0.62	0.64	0.31	0.59	0.63	0.66	0.51	0.58	0.47	0.47	0.56	–				
13	0.55	0.56	0.39	0.57	0.58	0.56	0.58	0.48	0.57	0.60	0.65	0.55	–			
14	0.56	0.58	0.40	0.57	0.57	0.59	0.61	0.52	0.52	0.58	0.62	0.56	0.71	–		
15	0.56	0.58	0.24	0.58	0.58	0.52	0.49	0.47	0.53	0.46	0.62	0.51	0.60	0.58	–	
16	0.47	0.51	0.46	0.54	0.55	0.53	0.62	0.43	0.54	0.65	0.61	0.53	0.65	0.68	0.59	–

### 3.2 Exploratory factor analysis

Our EFA process examined up to six factors (see Table 4 for model fit statistics). For the first five factors, each subsequently added factor was a significant improvement in model fit; however, adding the sixth factor did not significantly improve model fit. The two-factor and three-factor models both included two cross-loading items, the four-factor model had four cross-loading items, and the five-factor model had three cross-loading items. Although model fit continued to improve, the eigenvalues suggested that only the 1- and 2-factor models should be retained. The correlation between the two factors was 0.78. We, therefore, moved forward with testing the two-factor EFA model using CFA, excluding the two cross-loading items (see Table 5).

**Table 4:** Fit indices for competing EFA models of the pet-RFL items.

Model	X^2^	*df*	X^2^ Δ	*df Δ*	RMSEA	CFI	TLI	Eigenvalue
1-factor	424.86	104			0.102	0.966	0.961	10.71
2-factor	176.25	89	153.30^***^	15	0.057	0.991	0.988	1.16
3-factor	132.87	75	43.81^***^	14	0.051	0.994	0.990	0.66
4-factor	102.81	62	31.73^**^	13	0.047	0.996	0.992	0.54
5-factor	77.39	50	27.13^*^	12	0.043	0.997	0.993	0.49
6-factor	61.51	39	18.60	11	0.044	0.998	0.993	0.45

**Table 5:** Factor loadings for the 2-factor exploratory factor analysis (EFA) model (*N* = 299).

Items	Factor 1	Factor 2
1	**1.08** ^*^	−0.17^*^
2	**0.94** ^*^	0.001
3	−0.004	**0.65** ^*^
4	**0.75** ^*^	0.14
5	**0.86** ^*^	0.10
6	**0.71** ^*^	0.18^*^
7	0.39^*^	0.47^*^
8	**0.73** ^*^	−0.002
9	0.07	**0.68** ^*^
10	−0.09	**0.88** ^*^
11	0.24	**0.65** ^*^
12	0.52^*^	0.33
13	0.12	**0.81** ^*^
14	0.11	**0.78** ^*^
15	0.20	**0.66** ^*^
16	−0.04	**0.93** ^*^

Bolded items indicate items retained. ^*^p < 0.05.

### 3.3 Confirmatory factor analysis

Although the EFA identified a 2-factor model, we also tested and compared theoretically informed alternative models, including a unidimensional model, a 4-factor model, and a higher-order 4-factor model. The 2-factor EFA model aligned with prior research on reasons for living and the interpersonal psychological theory of suicide. One EFA factor included items that indicated belongingness [e.g., *I want to continue making memories with my pet(s)*] and feelings of responsibility and love for their pets [e.g., *I have a commitment to my pet(s*)]. The second EFA factor included items characterized by worry about burdening others [e.g., *It would not be fair to leave my pet(s) for others to take care of* ] and moral obligation [e.g., *I would let my pet(s) down if I chose to not be here anymore*].

To evaluate whether a more differentiated conceptual structure provided improved theoretical or empirical fit, we also specified a theoretically informed 4-factor model grounded in prior reasons for living research and the interpersonal theory of suicide. The first factor, *Responsibility to Pets*, included items 1, 2, 9, and 14 and aligns with the RFL Responsibility to Family Subscale. The second factor, *Pet Dependence and Welfare Risk*, included items 4, 5, 10, 11, 13, and 15. These items were grouped together to align with the RFL Child-related Concerns subscale, in that they focus on concerns for the pet's wellbeing as a reason for living. The third factor, *Emotional Attachment to Pets*, included items 6, 8, and 12, and is a counter to perceived thwarted belongingness and aligns with the RFL survival and coping beliefs subscale by reflecting companionship, meaning, and purpose. Finally, the fourth factor, *Anticipated Emotional Harm to the Pet*, included items 3, 7, and 16. This factor was formed to include items that approximate moral obligation and perceived burdensomeness by avoiding emotional harm to pets.

The unidimensional model demonstrated adequate model fit (CFI = 0.96, TLI = 0.95, RMSEA = 0.12, SRMR = 0.05), the 2-factor EFA model demonstrated good model fit (CFI = 0.98, TLI = 0.98, RMSEA = 0.08, SRMR = 0.03), and the 4-factor model (CFI = 0.96, TLI = 0.96, RMSEA = 0.11, SRMR = 0.05) and the higher-order 4-factor model demonstrated adequate fit (CFI = 0.96, TLI = 0.96, RMSEA = 0.11, SRMR = 0.05). Since the unidimensional and 4-factor models were nested, the difference test indicated that the additional complexity of the 4-factor model was a significant improvement in model fit (X^2^Δ = 203.19, *p* < 0.001). The difference test comparing the unidimensional model and the higher-order 4-factor model indicated that the higher order model fit better (X^2^Δ = 180.88, *p* < 0.001); however, comparing the higher-order 4-factor model with the 4-factor model resulted in a significant decrease in model fit (X^2^Δ = 13.28, *p* = 0.001). Therefore, we proceeded with comparing the 2-factor EFA model with the 4-factor CFA model. The factors in the 4-factor model were highly correlated (rs = 0.86–0.95) and the correlation between the two EFA-model factors was 0.85 in the full sample. Standardized factor loadings for the 2-factor EFA model ranged from 0.63 to 0.95, and ranged from 0.65 to 0.94 in the 4-factor model (see Table 6). Overall, both models demonstrated good model fit; however, the 2-factor EFA model had better fit compared to the 4-factor model based on ΔCFI (ΔCFI > 0.01) and greater parsimony. Therefore, subsequent analyses were conducted using the 2-factor EFA model.

**Table 6:** Standardized factor loadings of the final 4-factor CFA and 2-factor EFA models.

Pet-RFL item	CFA model				EFA model	
	Resp	Dep	Emot	Harm	Factor 1	Factor 2
1	0.93				0.93	
2	0.94				0.95	
3				0.65		0.63
4		0.90			0.92	
5		0.92			0.93	
6			0.89		0.85	
7				0.84		
8			0.77		0.74	
9	0.74					0.75
10		0.81				0.82
11		0.84				0.87
12			0.85			
13		0.84				0.86
14	0.88					0.87
15		0.78				0.81
16				0.85		0.84

CFA model factors were: Resp, Responsibility to Pets; Dep, Pet Dependence and Welfare Risk; Emot, Emotional Attachment to Pets; and Harm, Anticipated Emotional Harm to the Pet.

### 3.4 Measurement invariance testing

We first tested configural invariance using multiple group analyses across gender modality, race/ethnicity, relationship status, parental status, age, and pet type in separate models (see Table 7). All models imposing the same factor structure across groups fit the data adequately, providing support for configural invariance across groups using the 2-factor EFA model. We also found support for strong measurement invariance after imposing factor-loading and threshold constraints across all groups. This provides evidence that the latent factor means can be meaningfully compared across all of the sociodemographic groups we examined.

**Table 7:** Fit indices for the 2-factor EFA model and measurement invariance indices.

Model	X^2^	*df*	X^2^ Δ	*df Δ*	*p*	RMSEA	CFI	TLI
*2-factor EFA, full sample*								
	542.74	76				0.08	0.984	0.981
*Multiple group by gender modality (gender minority/cisgender men and women)*								
Configural invariance	640.88	228				0.08	0.986	0.984
Scalar invariance	645.00	276	59.82	48	0.118	0.07	0.988	0.988
*Multiple group by race/ethnicity (White, Hispanic, Black, Other)*								
Configural invariance	706.83	304				0.08	0.987	0.984
Scalar invariance	759.20	376	76.78	72	0.328	0.07	0.988	0.988
*Multiple group by relationship status (single, in a relationship)*								
Configural invariance	625.74	152				0.08	0.985	0.982
Scalar invariance	641.21	176	27.46	24	0.284	0.08	0.985	0.985
*Multiple group by child status (no children, has children)*								
Configural invariance	618.96	152				0.08	0.984	0.981
Scalar invariance	640.61	176	35.02	24	0.068	0.08	0.985	0.984
*Multiple group by age group (emerging adulthood, early adulthood, middle adulthood, older adulthood)*								
Configural invariance	784.87	304				0.08	0.985	0.982
Scalar invariance	845.64	376	84.64	72	0.146	0.07	0.985	0.986
*Multiple group by pet type (cat only, dog only, cat and dog)*								
Configural invariance	669.23	228				0.08	0.984	0.981
Scalar invariance	692.64	276	42.34	48	0.703	0.07	0.985	0.985

### 3.5 Construct validity testing with mental health and suicidality measures

Next, we evaluated internal consistency for each factor. McDonald's omega was excellent for both factors: factor 1 = 0.91 and factor 2 = 0.90.

We tested construct validity by examining associations between the established 2-factor EFA model and mental health and suicidality measures and the original RFL measure. We examined associations with and without covariates included in the model (see Table 8). There were a few differences in statistical significance between the conditional and unconditional models. To account for the effects of covariates, the conditional model results are presented below; however, the unconditional model results can be found in Table 8. We anticipated that higher mean scores on the Pet-RFL would be associated with lower levels of mental health problems and suicidality and lower likelihood of a suicide attempt history. The results partially supported our hypotheses. Higher scores on the EFA model factor 1 were associated with lower suicidal ideation (BSS, SIDAS), fewer depressive and stress symptoms (DASS-21), higher scores on the RFL Survival and Coping Beliefs and Moral Objections subscales, and a higher overall RFL score. Higher scores on the EFA model factor 1 were also associated with lower odds of lifetime suicide attempt history. In contrast, higher scores on factor 2 of the EFA-derived model (characterized by worry about leaving pet caregiving to others and moral obligation to pets) were associated with greater suicidal ideation (BSS, SIDAS), more depressive and stress symptoms (DASS-21), higher scores on the RFL Responsibility to Family and Fear of Suicide subscales, and lower scores on the RFL Moral Objections subscale. Higher scores on factor 2 of the EFA-derived model were also associated with greater odds of lifetime suicide attempt history.

**Table 8:** Associations between the EFA factors and DASS, BSS, SIDAS, and RFL subscales (*N* = 962).

Subscales	Unconditional model				Conditional model (*n* = 838)			
	Factor 1		Factor 2		Factor 1		Factor 2	
	B	*p*	B	*p*	B	*p*	B	*p*
DASS depression	**−1.93**	**0.025**	2.37	0.058	**−2.84**	**0.001**	**3.22**	**0.013**
DASS anxiety	−0.08	0.926	−0.04	0.975	−0.70	0.412	0.68	0.583
DASS stress	−1.35	0.103	1.98	0.101	**−2.16**	**0.014**	**2.92**	**0.023**
BSS total score	**−1.02**	**0.030**	**1.71**	**0.011**	**−1.09**	**0.027**	**1.64**	**0.019**
SIDAS total score	**−2.50**	**0.007**	**2.79**	**0.039**	**−2.96**	**0.002**	**3.01**	**0.033**
RFL survival and coping beliefs	**0.30**	**< 0.001**	−0.16	0.163	**0.36**	**< 0.001**	−0.18	0.080
RFL responsibility to family	0.02	0.823	**0.35**	**0.002**	0.10	0.220	**0.37**	**0.001**
RFL child-related concerns	0.17	0.268	−0.11	0.620	0.24	0.055	−0.04	0.828
RFL fear of suicide	**−0.23**	**0.034**	**0.50**	**0.002**	**−0.24**	**0.037**	**0.59**	**0.001**
RFL fear of social disapproval	0.11	0.473	0.09	0.662	0.14	0.385	0.16	0.486
RFL moral objections	**0.49**	**0.002**	**−0.56**	**0.015**	**0.42**	**0.010**	**−0.46**	**0.047**
RFL mean score	**0.19**	**0.007**	−0.01	0.917	**0.23**	**< 0.001**	0.01	0.917
	OR	95% CI	OR	95% CI	OR	95% CI	OR	95% CI
Suicide attempt (=yes)	0.92	0.82, 1.02	**1.44**	**1.04, 1.98**	**0.86**	**0.75, 0.98**	**1.70**	**1.16, 2.49**

Bolded values indicate statistical significance. Estimates provided are unstandardized coefficients. Due to the binary nature of the suicide attempt variable, it was examined using logistic regression and the coefficients were exponentiated to odds ratios for interpretability. 95% confidence intervals (CI) are presented instead of *p*-values for these results, and indicate statistical significance if the confidence interval does not include 1. The conditional model includes covariates.

## 4 Discussion

This study describes the psychometric development and validation of the newly developed Pet-Specific Reasons for Living Scale (Pet-RFL), extending the original Reasons for Living framework to include companion animals. Findings provide strong initial evidence that pet-related reasons for living are measurable, internally consistent, and clinically meaningful. Results also indicated that pet-related reasons for living are not unidimensional; they appear to cluster into at least two distinct motivational domains with differing associations to mental health and suicidality.

Across exploratory and confirmatory analyses, results converged on a parsimonious two-factor structure reflecting (1) pet-related belongingness, emotional connection, and sense of loving responsibility toward pets (e.g., desire to continue making memories with pets; commitment to pets), and (2) anticipated harm to pets or others if the person were to end their life (e.g., it would be unfair to leave the pet for others to care for; one would let the pet down by no longer being alive). This two-factor solution demonstrated strong factor loadings, excellent internal consistency, and measurement invariance across gender identity/modality, race/ethnicity, age/developmental stage, parental status, and pet type, supporting its utility for comparative research across diverse populations.

Importantly, the study's findings suggest that pet-related reasons for living may arise through at least two qualitatively different pathways: one rooted in connection and meaning, and another rooted in fear of harming one's pet or burdening future pet caregivers. The first factor captures companionship, future-oriented meaning, relational continuity, and commitment to the ongoing care of a dependent animal. From the perspective of the interpersonal psychological theory of suicide (35), these qualities may counter thwarted belongingness and perceived burdensomeness by reinforcing felt connection, purpose, and relational significance. This factor's overlap with caregiving and commitment also maps conceptually onto the original RFL domains of survival and coping beliefs and responsibility to family (8). In contrast, the second factor appeared to capture a more complex and potentially distress-linked pattern of pet-related reasons for living that may simultaneously function as both a deterrent to suicide and an indicator of distress related to concerns about pets and caregiving responsibilities. Rather than reflecting belongingness and adaptive commitment, these items centered on worry about leaving pets behind, concern that others would be burdened by the pet's care, and fear of emotionally harming a dependent animal. In essence, the study findings suggest that pet-related reasons for living can be seen as life-oriented or harm-avoidant, a conceptualization consistent with approach-avoidance models of motivation that distinguish between tendencies to approach desired outcomes and tendencies to avoid undesired outcomes (69, 70). For example, Higgins (70) identifies motivations based on striving toward gains and aspirations, vs. motivations based on preventing losses, fulfilling obligations, and avoiding negative outcomes.

The study's construct validity findings further underscored the approach-avoidance distinction in motivations. Higher scores on the Pet-RFL's first (i.e., life-oriented) factor were associated with lower suicidal ideation across both the BSS and SIDAS; lower depressive and stress symptoms; stronger endorsement of broader survival and coping beliefs, as measured by the Reasons for Living Inventory (8); and lower odds of a prior suicide attempt in the EFA model controlling for covariates. These findings extend prior qualitative literature [e.g., (6, 17, 31, 36)] in which individuals describe pets as “reasons to stay alive.” In contrast, higher scores on the Pet-RFL's second (i.e., harm-avoidant) factor were associated with greater suicidal ideation, greater likelihood of lifetime suicide attempt history, greater depression and stress symptoms, and stronger endorsement of fear-based and obligation-related reasons for living, when adjusting for covariates. These associations align with prior qualitative reports of individuals staying alive because they could not bear the idea of abandoning, harming, or failing an animal who depends on them (6, 31, 36, 71, 72). Along the same lines, prior research has found that being a parent can be a reason for living (73, 74) while paradoxically other research suggests that distress related to parenting can be related to suicide attempts (75). This distinction mirrors the broader human-animal interaction literature, which has increasingly emphasized that bonds with companion animals may function as both sources of resilience and sites of vulnerability depending on relational and contextual conditions (76, 77). The association between this factor and the RFL Responsibility to Family subscale further suggests that obligation-based concerns about pets may overlap with broader concerns about loved ones and dependents. Similar to family-related reasons for living, worries about abandoning a dependent animal or burdening others with caregiving responsibilities may function as deterrents to suicide while simultaneously reflecting distress and concern about anticipated harm to others.

### 4.1 Clinical and practice implications

The Pet-RFL offers a structured way to assess pet-related reasons for living that may otherwise be missed in standard suicide risk assessments. For many individuals, companion animals represent highly salient sources of connection, purpose, routine, caregiving responsibility, and emotional support that function alongside more commonly assessed protective factors such as family relationships, future goals, and social support (8, 31). Given that companion animal ownership is now widespread and, in some age groups, rivals or exceeds rates of parenthood, partnership, or marriage, omitting pet-related commitments from traditional reasons-for-living measures may overlook one of the most common contemporary forms of daily caregiving and attachment (78, 79).

At the same time, bonds with companion animals should not be viewed as uniformly protective. The identification of two distinct pet-related pathways—one reflecting connection, meaning, and caregiving, and the other reflecting obligation, guilt, and concern about burden—indicates that clinicians should look beyond mere pet ownership to the specific meanings, responsibilities, and concerns individuals attach to their relationships with companion animals. Statements such as “My dog gives me a reason to get up each morning” may differ meaningfully from statements such as “I can't die because my dog would go to a shelter and maybe get euthanized.” Both may represent reasons for living, yet they may signal different levels of hopelessness, distress, and clinical need.

Suicide risk assessments may therefore benefit from nuanced inquiry into whether pets are experienced primarily as sources of connection and meaning, sources of obligation and guilt, or both. Incorporating pet-related questions into routine mental health assessments, including tools such as the Pet-RFL, may help identify important sources of purpose and emotional connection as well as areas of distress, such as fears about abandoning a pet and burdening others with pet care in the event of suicide. This more nuanced understanding can strengthen suicide risk assessment and support more individualized, context-sensitive care.

These findings are also relevant for suicide safety planning. Pet-related activities such as feeding, walking, grooming, playing, or spending quiet time with an animal may serve as concrete grounding, distraction, mindfulness, or self-compassion strategies. Clinicians might also explore how pets provide humor, loving touch, routine, supportive presence, or a sense of being needed. However, these same responsibilities could aggravate distress when individuals feel overwhelmed by obligation, guilt, financial strain, housing instability, or fear about what would happen to their pet during a crisis. Clinicians should therefore proactively plan around pet-related concerns during hospitalization, residential treatment, housing transitions, sudden pet loss, pet illness, euthanasia decisions, or other crises (6, 19).

The study's implications extend beyond individual assessment to veterinary social work, integrated behavioral health, shelter settings, and broader suicide prevention efforts. Concerns about who will care for a pet may function not only as salient reasons for living, but also as barriers to accessing inpatient or residential mental health treatment, making pet care planning a potentially important component of crisis intervention and discharge planning (19, 80, 81). Veterinary social workers are particularly well positioned to assess pet-related concerns during high-stress situations such as serious pet illness, euthanasia decisions, financial strain, behavioral concerns, potential relinquishment, or mental health crises in which fears about a pet's future care may intensify distress or complicate treatment access. They may also help facilitate pet care planning and support arrangements during periods of mental health crisis, including temporary fostering, caregiving continuity, or related pet-related needs.

More broadly, the findings support greater consideration of pet-related concerns within suicide prevention, crisis response, and behavioral health service delivery. Because concerns related to pet welfare, caregiving continuity, and burden on others emerged as salient dimensions of pet-related reasons for living, pet-inclusive supports and care-planning approaches may help reduce treatment barriers and distress during psychiatric hospitalization, housing instability, or other crises (81–84).

### 4.2 Limitations and future directions

Several limitations should be considered when interpreting these findings. First, the cross-sectional design precludes conclusions about temporal or causal relationships between pet-related reasons for living and suicidality. For example, it cannot be determined whether pet-related belongingness reduces suicidal ideation, whether individuals with less suicidal ideation are more able to experience pet relationships positively, or whether bidirectional processes are involved. Longitudinal studies are needed to determine whether the life-oriented belongingness pathway prospectively predicts reduced suicide risk and whether the harm-avoidant obligation/burden pathway reflects co-occurring distress, crisis persistence, delayed action, or a distinct deterrent process. Second, the use of an online Prolific sample of U.S. adults may limit generalizability to clinical populations, as well as to culturally diverse groups outside the United States. Replication in psychiatric, inpatient, youth, older adult, and structurally marginalized populations will be important for establishing broader clinical utility. Third, all constructs were assessed through self-report, raising the possibility of recall bias, social desirability, mood-state effects, and shared method variance, particularly given the conceptual overlap between current distress severity and obligation-based, harm-avoidant reasons for living. Research incorporating clinical interviews, ecological momentary assessment, or prospective follow-up could help clarify the stability and behavioral relevance of Pet-RFL scores.

Additional limitations relate to scale development and validation. Although the Pet-RFL items were newly developed, they were informed by the structure and phrasing of the original Reasons for Living Inventory to maintain conceptual continuity. As a result, some aspects of the observed factor structure may partially reflect shared wording or format characteristics inherited from the original measure rather than entirely distinct latent constructs. Future psychometric refinement should evaluate alternative item wording, including separate singular and plural forms for respondents with one vs. multiple companion animals, and use cognitive interviewing to test whether the same latent structure emerges and whether these adaptations improve readability or psychometric performance. In addition, collapsing the RFL's original six response categories into three categories to address sparse cell frequencies likely reduced response nuance and may have obscured meaningful gradations in endorsement intensity. Larger samples with greater suicide risk severity may allow testing of the full response structure and more advanced item-level psychometric modeling, such as item-response-theory approaches. Finally, although the factor structure was explored in a random subsample and confirmed in the larger analytic sample, all analyses were derived from the same broader dataset. Independent replication is especially important for the second factor, whose interpretation as a distress-linked, co-occurring obligation process remains theoretically compelling but provisional.

## 5 Conclusion

By extending the original reasons for living framework to include companion animals, this psychometric study of the Pet-Specific RFL demonstrated that pet-related beliefs and values are a clinically relevant and psychometrically measurable domain of deterrents to suicide. The two-factor structure further suggests that pets may represent important reasons for living for many individuals, but in different ways. Pets appear to foster belongingness and meaning, and they also may give rise to harm-avoidant fears and obligations. Recognizing this distinction may improve measurement and clinical assessment of the roles that companion animals can play in suicidal crises. Suicide prevention is most effective when it reflects the lived realities of those it seeks to serve; for many individuals, those realities include profound and life-affirming bonds with companion animals.

## Data Availability

Data may be available upon request. Requests to access this data should be sent directly to smcdon27@utk.edu.
